# Comments on “Assessment of changes in the liver of pregnant female rats and their fetuses following monosodium glutamate administration” by Gad El-Hak et al., http://doi.org/10.1007/s11356-021–13,557-7

**DOI:** 10.1007/s11356-022-23858-0

**Published:** 2022-10-28

**Authors:** Shintaro Yoshida, Masanori Kohmura

**Affiliations:** International Glutamate Technical Committee (IGTC), National Press Building 529, 14Th Street, Suite 1280, Washington, DC 20045 USA

To the Editor,

In the article entitled “Assessment of changes in the liver of pregnant female rats and their fetuses following monosodium glutamate administration” by Gad EL-Hak et al. (2021), it is reported that forced oral administration of monosodium glutamate (MSG) from day 0 to day 20 of pregnancy induced biochemical, histological, and histochemical changes in the liver of maternal and fetal rats. The authors conclude that these study results indicate the risks of human consumption of MSG in pregnancy. In response to the authors’ conclusion, we would like to offer some comments on this study, and the safety of MSG used in foods.

In this study, the authors used pregnant rats that received MSG at a dose of 1 g/kg bw and stated that this dosage is estimated to correspond to a person consuming 0.3–1.0 g/day of MSG (Beyreuther et al. 2007). However, if this dose were to be applied to a person weighing 50 kg bw, it would be necessary to ingest 50 g of MSG per day. It is not at all consistent with the estimation of daily MSG consumption in humans and far exceeds the scope of a food additive. In addition, MSG was administered to rats through a gastric tube. Since MSG as a food additive is ingested with foods, it is clear that the route of administration in this study is also inappropriate for an assessment of the safety of MSG used as a food additive.

It is well established that almost all glutamate ingested with food is metabolized in the gut, serving primarily as an energy source for enterocytes, and cannot enter the circulation (Wu 1998; Reeds et al. 2000). Almost all of the dietary glutamate ingested with food was reported to be metabolized into CO_2_, lactate, alanine, or proline (56%, 13%, 12%, 3% of the dietary input, respectively) in the rat gut (Nakamura et al. 2013). It has also been demonstrated that dietary glutamate is intensively oxidized during the splanchnic first pass in healthy adults and preterm infants (Battezzati et al. 1995; Riedijk et al. 2007). Furthermore, it has been shown that normal dietary glutamate ingestion has only a negligible effect on blood glutamate concentration in humans (Stegink et al. 1985; Ghezzi et al. 1985; Reeds et al. 2000). It has also been clarified that even a high dose (100 mg/kg bw/day) of MSG added to food results in only a slight change in plasma glutamate levels in humans, which is within circadian variation (Tsai & Huang 1999), and that the basal glutamate concentration in humans is not affected by long-term MSG consumption (Tanphaichitr et al. 2000). Rodent studies have shown that feeding a diet containing MSG has little influence on blood glutamate levels and these studies suggest that the major nutrient in foods, metabolizable carbohydrate, is most effective in attenuating any rise in blood glutamate levels induced by oral administration of MSG (Fernstrom et al. 2002). Moreover, in the studies conducted under standard feeding conditions, rats with free access to water containing MSG did not show any rise in the blood concentrations of amino acids. Even if MSG is consumed in large amounts under these study conditions, blood levels of amino acids, including glutamate, were shown to be maintained at almost constant levels (Kondoh & Torii 2008; Nishigaki et al. 2018).

Citing some research reports about the effect of MSG ingestion on the fetus, the authors argue that glutamate can pass through the placenta and enter the fetal circulation, and maintain, based on the results of this study, that maternal administration of MSG also causes adverse effects on the fetal and maternal liver. However, in humans, glutamate levels in both the umbilical artery and vein are much higher than that in maternal blood (Young & Prenton 1969; Zlotnik et al. 2012). And it has been demonstrated that the fetal liver is the primary site for glutamate production and glutamate is supplied to the placenta mainly from the fetal circulation (Battaglia 2000). This means that since the placenta obtains glutamate from the fetal liver and actively metabolizes it, glutamate from the maternal circulation cannot penetrate the placenta. In addition, it is reported that infusion of MSG into pregnant rhesus monkeys, resulting in a 10- to 20-fold increase in maternal blood glutamate levels, could not raise blood glutamate levels in the fetus (Stegink et al. 1975; Pitkin et al. 1979). Another in vitro perfusion study using a human placenta indicated that the placenta served as an effective metabolic barrier to the transfer of glutamic acid (Schneider et al. 1979). These studies suggest that the placenta actually extracts its glutamate from the fetal liver as an energy source and not from the maternal circulation (Battaglia, 2000). This shows that the placenta is virtually impermeable to glutamate and acts as a barrier to the transfer of maternal glutamate to the fetus. Taken together with the scientific fact that ingestion of MSG cannot raise the maternal blood glutamate level, it is evident that MSG added to food cannot exert any influence on the human fetus.

MSG, a sodium salt of the naturally occurring glutamic acid, is widely used as a food additive to leverage the flavor of foods and is always ingested with food. Since 1958, glutamate has been listed as a generally recognized as safe (GRAS) substance by the US Food and Drug Administration (FDA). The Joint FAO/WHO Expert Committee (JECFA) also evaluated the safety of MSG in 1970, 1973, and 1987. Finally, JECFA concluded that the total dietary intake of glutamate did not represent a hazard to health and that it is not necessary to establish a numerically acceptable daily intake for humans, including pregnant women and infants. In the rat studies of MSG conducted at doses and via an administration route applicable to normal human dietary intake and glutamate exposure, it was also demonstrated that MSG could not cause any changes in the biochemical parameters associated with liver function (Owen et al. 1978; Kolawole 2013). The study reported by El-Hak et al. does not employ appropriate methods that reflect how MSG is actually used in food and the study result misleads readers about the safety of MSG. We argue that the experimental methodology, including dose and routes, should be designed to be scientifically valid when assessing the safety of MSG in food.
